# DLS-Derived Apparent Mobility as a Formulation-Relevant Descriptor of Thermoresponsive Methylcellulose Gelation and Hysteresis

**DOI:** 10.3390/gels12090766

**Published:** 2026-08-26

**Authors:** Franz Miller Branco Ferraz, Christina Reichart, Laura Kainz, Christian Moitzi

**Affiliations:** Anton Paar GmbH, Anton-Paar-Strasse 20, 8054 Graz, Austria; christina.reichart@anton-paar.com (C.R.); laura.kainz@anton-paar.com (L.K.); christian.moitzi@anton-paar.com (C.M.)

**Keywords:** methylcellulose, thermoresponsive gels, dynamic light scattering, apparent diffusion coefficient, sol–gel transition, thermal hysteresis, polymer association, turbidity, oscillatory rheology

## Abstract

Methylcellulose is a thermoresponsive polymer that undergoes thermally induced association and gelation upon heating, with behavior strongly influenced by thermal history, concentration, and ionic environment. In this work, dynamic light scattering (DLS) was used beyond conventional particle size analysis to monitor the temperature-dependent mobility of methylcellulose solutions through the apparent diffusion coefficient, complemented by transmittance and oscillatory rheology. For a 0.1 wt.% methylcellulose solution, rheology showed a sol–gel transition during heating between approximately 50 and 60 °C, but no complete gel–sol transition during cooling, indicating pronounced thermal hysteresis. Transmittance and DLS confirmed this path dependence while revealing different recovery behavior: optical turbidity recovered near 38–40 °C, whereas apparent mobility recovered at slightly lower temperatures, around 30–35 °C. A simple Arrhenius-type model described sol-state mobility but not the full heating cycle. Therefore, a two-state phenomenological model was introduced, representing DLS-derived mobility as weighted sol-like and gel-like contributions. The model captured mobility loss, recovery, and a hysteresis window of about 22 °C. Overall, DLS-derived apparent mobility provides a useful descriptor of methylcellulose association, dissociation, and hysteresis, complementing rheology and turbidity measurements.

## 1. Introduction

Methylcellulose (MC) is a nonionic, partially methyl-substituted cellulose ether that combines aqueous processability at low temperature with thermally induced association and gelation upon heating. This unusual inverse thermoresponsive behavior distinguishes MC from many conventional polymer gels and underlies its use as a thickener, binder, stabilizer, film-forming material, extrusion aid, and thermoresponsive matrix in food, pharmaceutical, biomedical, construction, coating, and other industrial formulations [1,2]. The balance between the remaining hydrophilic hydroxyl groups and the more hydrophobic methoxy substituents enables MC to dissolve in cold water, while increasing the temperature progressively reduces the quality of water as a solvent and promotes intermolecular association [1,2,3]. Although this behavior is commonly described as thermoreversible gelation, heating and cooling generally do not follow coincident paths. Gel formation, structural relaxation, and dissolution may occur over substantially different temperature ranges, producing pronounced thermal and mechanical hysteresis.

Rheological, calorimetric, spectroscopic, and light scattering studies have described MC thermogelation as a multistage process involving disruption of polymer hydration, exposure of hydrophobic methoxy-rich regions, progressive intermolecular association, formation of polymer-rich heterogeneities, and eventual development of a mechanically connected network [3,4,5,6,7,8,9,10]. These studies established that MC gelation cannot be represented adequately as a single instantaneous transition. Instead, gradual pre-gel association may precede a more rapid increase in turbidity and elasticity. Later studies combining rheology, turbidity, small-angle neutron or X-ray scattering, static light scattering, and cryogenic transmission electron microscopy substantially refined this picture [11,12,13,14,15,16,17]. Thermally associated MC chains form semiflexible fibrils with characteristic diameters of approximately 14–20 nm, which connect and percolate into a macroscopic network. The fibril diameter is comparatively insensitive to polymer concentration and molar mass, whereas fibril length, network connectivity, and gel modulus depend more strongly on chain length and formulation conditions [12,13,14,15,16]. Wide- and mid-angle X-ray scattering further indicate that thermally formed fibrils contain relatively ordered or semicrystalline regions interspersed with less organized domains [17]. The persistence of these fibrillar structures during cooling has been directly associated with rheological hysteresis and incomplete structural recovery [13]. Recent experimental and molecular simulation studies continue to refine the chain-level description of MC association, including conformational rearrangement, hydrogen bond redistribution, hydrophobic interactions, chain packing, degree of substitution, and substitution pattern effects [18,19]. The current state of knowledge therefore supports a multiscale and multistage mechanism rather than a single equilibrium sol–gel event.

MC thermogelation is also strongly dependent on formulation and measurement history. Polymer concentration affects intermolecular contact probability, fibril connectivity, post-gel mobility, and mechanical strength and generally shifts the apparent gelation temperature as the system moves from dilute to semidilute and concentrated regimes [9,10,11,20]. Molar mass influences chain relaxation, fibril length, network connectivity, and gel modulus, even when the characteristic fibril diameter remains comparatively unchanged [14,15,16]. The degree and distribution of methoxy substitution determine the balance between hydration and hydrophobic association and can alter both the gelation temperature and the assembled fibril structure [5,17,19]. Added salts can substantially modify the gelation response, with the direction and magnitude of the effect depending on ion identity and concentration [17,21,22,23,24]. Thermal and mechanical protocols are similarly important: MC gelation can depend strongly on heating rate, whereas dissolution during cooling may show a different and often weaker rate dependence [11]. Applied shear or oscillatory stress can also alter association kinetics and the final network structure [25]. Differential scanning calorimetry and time-domain nuclear magnetic resonance have further demonstrated that dehydration, association, fibril formation, and water redistribution develop over finite and potentially overlapping time scales [26,27]. Thus, an experimentally reported “gelation temperature” is not an intrinsic formulation constant unless the thermal history, concentration, salt composition, measurement time, and mechanical protocol are also specified.

MC thermogelation is conventionally characterized using oscillatory rheology, often through changes in the storage modulus (G′), loss modulus (G″), and loss factor (tan(δ) = G″/G′). The temperature at which G′ exceeds G″ is frequently used as an operational gelation marker. However, a modulus crossover does not necessarily represent a unique critical gel point, particularly when both moduli change nonmonotonically or when no reverse crossover is observed during cooling. More rigorous rheological criteria based on Winter–Chambon scaling and frequency-independent tan(δ) may provide stronger evidence of critical gelation but require measurements at multiple frequencies and are not always applicable to rapidly evolving or strongly heterogeneous physical gels [28]. Turbidity or transmittance measurements provide a complementary optical marker by detecting the formation of polymer-rich domains and scattering heterogeneities [11,23,29,30]. Calorimetry probes the net enthalpic response associated with dehydration and association [21,22,26], whereas SANS, SAXS, WAXS, static light scattering, and cryo-TEM provide structural information over molecular-, fibrillar-, and network-length scales [12,13,14,15,16,17]. Time-domain NMR probes changes in polymer and water dynamics [27], and more recent optical imaging approaches have demonstrated that gelation and hysteresis can also be followed through temperature-dependent changes in sample appearance [31]. Because these techniques probe different aspects of the transition, they may identify different apparent transition temperatures.

Dynamic light scattering provides a particularly sensitive route for following changes in polymer dynamics during association and gelation. In an ideal dilute and ergodic system dominated by translational Brownian motion, the decay rate of the electric field autocorrelation function can be related to a translational diffusion coefficient and, under appropriate assumptions, to a hydrodynamic size. Those assumptions become progressively less secure when the sample contains interacting chains, polydisperse clusters, concentration fluctuations, static heterogeneities, or a percolated network. DLS studies of chemical and physical gels have shown that gelation may produce multiple relaxation modes, broad or nonexponential correlation functions, speckle patterns, frozen-in scattering contributions, and an ergodic-to-nonergodic transition [32,33,34]. Time-resolved DLS has consequently been used to identify gelation thresholds and critical dynamics, and agreement between DLS-derived and rheological gel points has been demonstrated for selected gel systems [34,35].

DLS has also contributed specifically to the understanding of MC solutions. In a seminal combined DLS, static light scattering, SANS, and rheological study, Kobayashi et al. identified two relaxation modes in semidilute MC solutions: a faster mode associated with cooperative diffusion and a slower mode attributed to pre-gel clusters [6]. Subsequent studies combining light scattering, rheology, and cryo-TEM connected changes in scattering and viscosity with thermally induced aggregation and network formation [30]. Static and dynamic light scattering measurements have also been used to examine the dilute solution conformation, hydrodynamic dimensions, and rod-like characteristics of MC-derived structures [36,37]. More recently, temperature-dependent DLS measurements have followed the development of large apparent hydrodynamic dimensions during heating and interpreted these changes in terms of progressive association and network development [38]. Collectively, these studies establish that DLS is sensitive to MC conformation, cooperative motion, clustering, aggregation, and gel formation.

Previous DLS studies have established that methylcellulose association and gelation affect relaxation modes, cluster dynamics, and apparent hydrodynamic dimensions [6,37,38]; however, to our knowledge, these observations have not been integrated into a common quantitative framework that describes the complete heating–cooling response, distinguishes mobility loss from mobility recovery, and yields comparable measures of transition width and thermal hysteresis across formulation conditions. The novelty of the present work is therefore not the use of DLS to detect methylcellulose association per se. Rather, the instrument-reported apparent diffusion coefficient is reinterpreted as a DLS-derived apparent mobility: a simple Arrhenius-type relation is used to establish the sol-like mobility baseline, and a two-state phenomenological model represents the measured response as weighted effective sol-like and gel-like dynamic contributions. This approach converts temperature-dependent DLS data into mobility scales, transition midpoints, transition widths, and heating–cooling hysteresis windows, which are compared directly with transmittance and oscillatory rheology markers and extended, within the experimentally calibrated formulation space, to polymer concentration and ionic strength effects using global parameterization. The resulting framework provides a formulation-relevant quantitative representation of methylcellulose gelation and dissociation that is not available from a conventional particle-sizing interpretation of DLS.

## 2. Materials and Methods

### 2.1. Sample Preparation

A commercial methylcellulose powder (Art. No. 339L, Carl Roth GmbH + Co. KG, Karlsruhe, Germany), with a nominal molar mass of 160 kg mol^−1^ and a viscosity grade of 12–18 mPa·s (2 wt.% aqueous solution at 20 °C), was used as received. The dry powder was characterized in terms of particle size and shape by dynamic image analysis using a Litesizer DIA 500 (Anton Paar GmbH, Graz, Austria) equipped with a Dry Jet dispersion unit. The particles exhibited an irregular morphology with a median equivalent diameter of 128.4 µm. Representative results are provided in Figure S1, and the corresponding particle size statistics are summarized in Table S1 in the Supplementary Information.

Aqueous methylcellulose solutions with polymer concentrations of 0.1 and 1.0 wt.% were prepared using pre-filtered deionized water, 0.9 wt.% NaCl, and 1.0 M KCl. The dry polymer was gradually added under stirring to reduce agglomeration and ensure homogeneous wetting. Dispersions were stirred at 450 rpm for 12 h at room temperature and stored at 4 °C for 12 h to promote hydration and dissolution. Before characterization, samples were equilibrated to the measurement temperature and visually inspected for macroscopic homogeneity.

### 2.2. Rheology

Oscillatory temperature sweeps were performed on 0.1 wt.% methylcellulose solutions using an MCR 503 rotational rheometer (Anton Paar GmbH, Graz,  Austria) to investigate the sol–gel transition and reversibility upon cooling. The rheometer was equipped with a CP50-1 cone-plate measuring system (Anton Paar GmbH, Graz, Austria) consisting of a 50 mm diameter cone with a cone angle of 1°. Temperature control was provided by a P-PTD 200/GL Peltier temperature device in combination with an H-PTD 220 Peltier temperature hood (Anton Paar GmbH, Graz, Austria). The hood provided additional thermal control around the upper measuring geometry and sample, helping to reduce temperature gradients during heating and cooling. The measurements were conducted to characterize the thermally induced sol–gel transition of the methylcellulose solutions and to evaluate its reversibility upon cooling.

The samples were subjected to small-amplitude oscillatory shear, in which a sinusoidal strain was applied according to

(1) $$\gamma \left(t\right)=\gamma_{0}\;\sin\left(\varpi t\right)$$

where $\gamma_{0}\;$ is the strain amplitude, and ϖ is the angular frequency. Measurements were carried out at a fixed strain amplitude of 1% and an angular frequency of 10 rad s^−1^.

Temperature sweeps were performed between 4 and 70 °C using a heating and cooling rate of 1 °C min^−1^. During the measurements, the storage modulus G′, loss modulus G″, and loss factor tan(δ) = G″/G′ were recorded as functions of temperature. These parameters were used to monitor viscous and elastic behavior during heating and cooling.

### 2.3. Transmittance and Dynamic Light Scattering

Temperature-dependent dynamic light scattering (DLS) and transmittance measurements were performed using a Litesizer DLS 501 with quartz cuvettes (Anton Paar GmbH, Graz, Austria). Each sample was subjected to a stepwise temperature series from 4 °C to 70 °C, with measurements collected at 2 °C intervals. At each temperature, the sample was equilibrated for at least 1 min before data acquisition. The measurement was then performed in manual quality mode using 100 runs with an acquisition time of 30 s per run, providing robust scattering statistics throughout the thermally induced transition.

The laser attenuation and focus were controlled automatically by the instrument software. Depending on the formulation, the solvent properties were defined as water, 1 M KCl, or 154 mM NaCl, corresponding to 0.9 wt.% NaCl.

Although the DLS experiments were performed using the particle size measurement mode, the analysis focused primarily on the apparent diffusion coefficient and transmittance. These parameters were selected because they are sensitive to mobility and turbidity changes during gelation, respectively.

The Kalliope 5.0.2 software reports an apparent diffusion coefficient derived from the measured relaxation rate. Because gelation introduces collective, aggregate, and network dynamics, the DLS-reported apparent diffusion coefficient is interpreted as DLS-derived apparent mobility rather than true translational diffusion.

Additionally, continuously monitored transmittance measurements were performed on 0.1 wt.% methylcellulose solutions as a function of time during 2 h at the same temperature, followed by temperature-jump experiments (from 70 °C to 60 °C, 70 °C to 50 °C and 70 °C to 40 °C) to investigate the kinetics of methylcellulose association and dissociation as a function of temperature. The decrease in transmittance indicates the development of turbidity associated with thermally induced methylcellulose association and gel formation.

## 3. Model Description

Two mobility-based models were used to describe temperature-dependent MC association and dissociation. The conceptual model describes one condition at a time, whereas the composition-dependent two-state model introduces empirical concentration and ionic strength dependencies.

Both models assume that the measured apparent diffusion coefficient reflects weighted contributions from a high-mobility sol-like state and a restricted gel-like state. The measured apparent diffusion coefficient is therefore represented as a weighted combination of sol-like and gel-like mobility contributions:

(2) $$D={f_{gel}\;D}_{gel}+\left(1-f_{gel}\right)\;D_{sol}$$

where $D_{sol}$ and $D_{gel}$ are the apparent diffusion coefficients of the sol-like and gel-like states, respectively, and $f_{gel}$ is the apparent gel-like mobility fraction. In this formulation, $f_{gel}$ = 0 represents a fully sol-like response, whereas $f_{gel}$ = 1 corresponds to a fully gel-like response.

### 3.1. Conceptual Model

The conceptual model was developed to describe one experimental condition at a time. It can be applied independently to a heating or cooling ramp of a given MC solution. In this model, the temperature dependence of the sol-like and gel-like mobility contributions is described using an Arrhenius-type expression:

(3) $$D_{i}(T)={D_{0}}_{i}{exp}^{\left(-\frac{{E_{a}}_{i}}{RT}\right)}$$

where i denotes either the sol-like or gel-like state. Here, ${D_{0}}_{i}$ is the pre-exponential factor, ${E_{a}}_{i}$ is the apparent activation energy that describes the temperature sensitivity of mobility in each state, R is the universal gas constant, and T is the absolute temperature.

The transition between the two states is described by a sigmoidal function:

(4) $$f_{gel}(T)=\frac{1}{1+{exp}^{\left(-\frac{T-T_{gel}}{∆T}\right)}}$$

where $T_{gel\to sol}$ is the characteristic temperature of the sol–gel or gel–sol transition, and ∆T describes the width or sharpness of the transition. Lower values of ∆T correspond to a sharper transition, whereas higher values indicate a broader conversion from sol-like to gel-like dynamics.

The conceptual model was fitted independently to each heating or cooling ramp. The transition temperature $T_{gel}$ represents sol–gel conversion during heating and gel–sol conversion during cooling; their difference quantifies hysteresis.

### 3.2. Composition-Dependent Two-State Model

While the conceptual model describes a single MC system, the composition-dependent two-state model was developed to estimate the apparent diffusion coefficient across multiple MC concentrations and ionic strengths. This model preserves the same two-state structure but introduces explicit concentration and ionic strength dependencies into the mobility functions and the transition temperature. The sol-like and gel-like mobility terms are modified to account for formulation-dependent changes in mobility:

(5) $$D_{i}(T,c,I)={D_{0}}_{i}{exp}^{\left(A(c,I)-\frac{{E_{a}}_{i}}{RT}\right)}$$

where A(c,I) represents the empirical correction for concentration and ionic strength in each dynamic state:

(6) $$A(c,I)=\;-\alpha c^{\upsilon }-\beta I$$

In the present formulation, concentration dependence is described by a power law term, $\alpha c^{\upsilon }$, while the ionic strength dependence is introduced through a linear term βI. These terms allow the model to account for the systematic reduction or modification of mobility caused by increasing polymer content and salt concentration.

The transition temperature is also expressed as a function of concentration and ionic strength:

(7) $$T_{gel}\left(c,I\right)=T_{0}-a\;ln\left(c\right)-b\sqrt{I}$$

where $T_{0}$ is the reference transition temperature, and a and b quantify the sensitivity of the gelation temperature to polymer concentration and ionic strength, respectively. The same sigmoidal expression used in the conceptual model is then evaluated using $T_{gel}\left(c,I\right)$, allowing the gel-like contribution to shift systematically with formulation conditions.

The composition-dependent model estimates DLS-derived apparent mobility as a function of temperature, polymer concentration, and ionic strength using one globally fitted parameter set. Unlike the conceptual model, which is fitted separately to individual datasets, the composition-dependent model is fitted globally to multiple datasets using one shared parameter set. This enables the investigated formulations to be described and compared within a common mobility-based framework.

### 3.3. Fitting Parameters and Physical Interpretation

Although both models are phenomenological, the fitting parameters have direct physical or semi-physical meaning. The pre-exponential factors ${D_{0}}_{sol}$ and ${D_{0}}_{gel}$ define the characteristic mobility scales of the sol-like and gel-like states, while the apparent activation energies ${E_{a}}_{sol}$ and ${E_{a}}_{gel}$ describe the temperature sensitivity of mobility in each state. The transition temperature $T_{gel}$, or $T_{gel}\left(c,I\right)$ in the composition-dependent two-state model, defines the effective sol–gel or gel–sol transition midpoint. The parameter ∆T describes the transition width, with smaller values corresponding to sharper transitions.

In the composition-dependent two-state model, α, υ, and β describe empirical effects of polymer concentration and ionic strength on the mobility terms, whereas a and b describe how these variables shift the transition temperature. These parameters therefore provide quantitative descriptors of mobility scale, temperature sensitivity, transition sharpness, and formulation-dependent trends within the calibrated formulation space.

### 3.4. Model Assumptions and Limitations

The model is phenomenological and describes DLS-derived apparent mobility rather than a thermodynamic phase transition. The apparent sol-like and gel-like fractions should be interpreted as effective dynamic contributions, not true mass fractions or equilibrium phase fractions.

The two-state representation does not explicitly resolve chain dehydration, cluster formation, fibrillar association, phase separation, or network percolation. Instead, these processes are captured only through effective transition and mobility parameters. The concentration and ionic strength terms describe trends across the measured formulation space but do not explicitly account for ion-specific effects, substitution pattern, molar-mass distribution, or non-equilibrium aging.

In this work, calibration refers to fitting the model parameters to measured DLS-derived apparent mobility. Interpolation refers to estimates for unmeasured conditions lying within the multidimensional formulation and temperature domain spanned by the calibration data. Validation refers to evaluation against independent data that were not used for parameter fitting. Extrapolation refers to estimates outside the calibration domain and should be interpreted with particular caution.

Despite these limitations, the framework provides a compact way to quantify and compare DLS-derived mobility changes during methylcellulose association and dissociation and to relate these changes to optical and rheological gelation markers.

### 3.5. Fitting Procedure

The model parameters were obtained by nonlinear regression using the Solver Add-in in Microsoft Excel. For each dataset, the DLS-derived apparent diffusion coefficients estimated by the model were calculated at the same temperatures used in the DLS experiments and compared with the corresponding measured apparent diffusion coefficients.

Optimization was performed by varying the model parameters to maximize the coefficient of determination, *R*^2^, between the measured and modeled apparent diffusion coefficients:

(8) $$R^{2}=1-\frac{\sum_{i=1}^{n}{\left(D_{exp,i}-D_{model,i}\right)}^{2}}{\sum_{i=1}^{n}{\left(D_{exp,i}-{\bar{D}}_{exp}\right)}^{2}}$$

where $D_{exp,i}$ is the experimentally measured DLS-derived apparent diffusion coefficient at the  i -th temperature, $D_{model,i}$ is the corresponding model-estimated value, and ${\bar{D}}_{exp}$ is the mean experimental apparent diffusion coefficient of the dataset. Thus, optimization of $R^{2}$ corresponds to maximizing the fraction of experimental variance explained by the model.

For the conceptual model, heating and cooling ramps were fitted independently. For the composition-dependent model, all selected formulation datasets were fitted simultaneously using one global parameter set and an average *R*^2^ objective:

(9) $$\bar{R^{2}}=\frac{1}{m}\sum_{j=1}^{m}{R^{2}}_{j}$$

where m is the number of experimental conditions included in the global fit. Parameter bounds ensured positive mobility pre-factors, positive transition widths, and realistic transition temperatures and apparent activation energies.

## 4. Results and Discussion

### 4.1. Rheological Properties and Determination of Gelation Temperature

**Figure 1:**
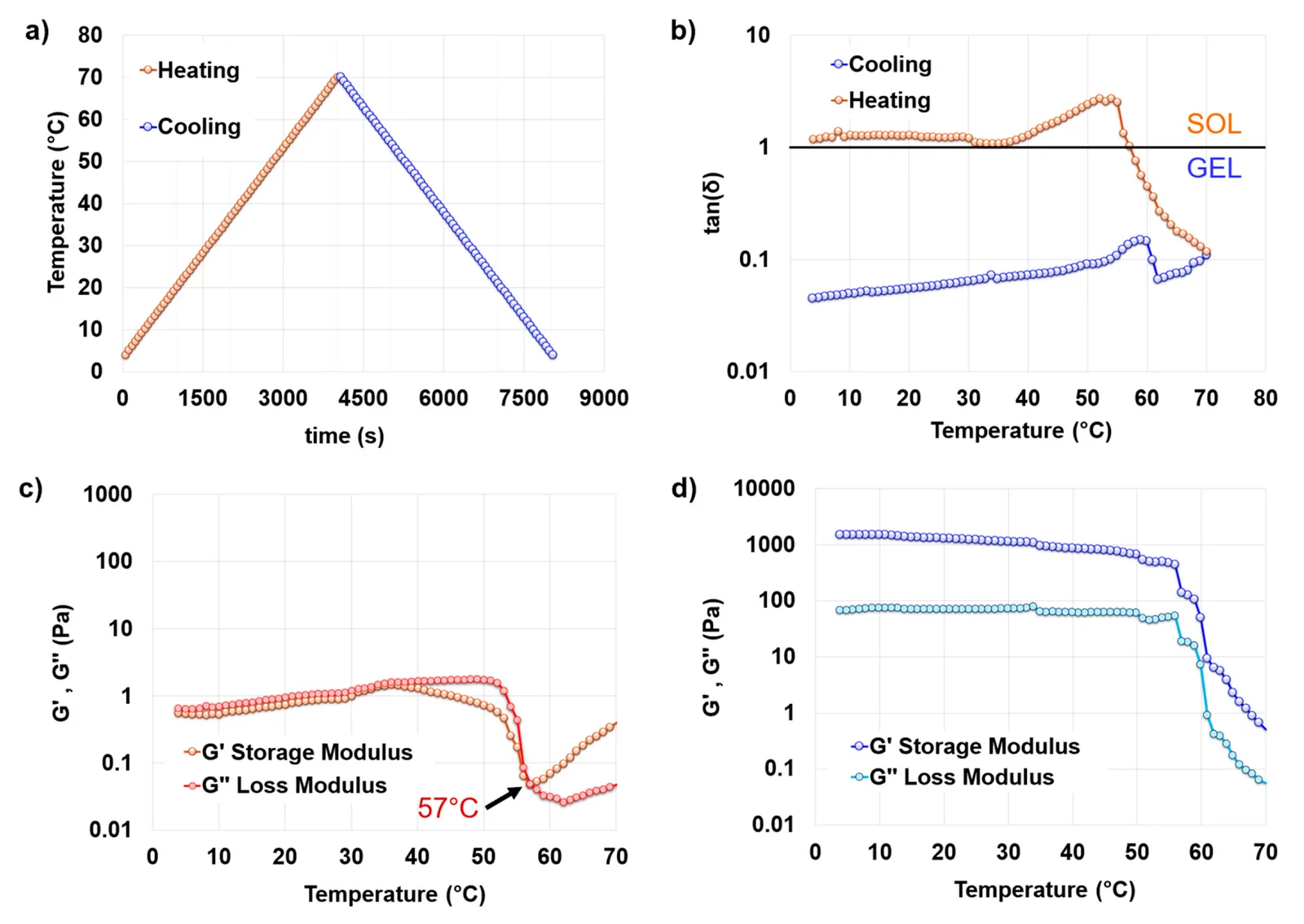
Thermo-rheological response of the investigated 0.1 wt.% methylcellulose solution during heating and cooling. (**a**) Thermal history applied during oscillatory measurements at 10 rad s^−1^ and 1% strain, with heating and cooling rates of 1 °C min^−1^. (**b**) Loss factor tan(δ), as a function of temperature during heating and cooling. (**c**) Storage modulus G′ and loss modulus G″ during heating. (**d**) Storage and loss moduli during cooling.

Oscillatory rheology revealed a pronounced thermoresponsive transition in the investigated 0.1 wt.% methylcellulose solution (Figure 1). At low temperature, tan(δ) was greater than 1, indicating predominantly viscous, sol-like behavior (Figure 1b). Upon heating, tan(δ) remained close to unity up to approximately 50–55 °C and then decreased sharply between 50 and 60 °C. The operational sol–gel transition occurred near 57 °C, where tan(δ) fell below 1 and G′ exceeded G″ (Figure 1c).

The heating response was not a simple monotonic increase in elasticity: both G′ and G″ decreased abruptly near 55 °C before G′ recovered and increased above approximately 57 °C (Figure 1c). This nonmonotonic response is compatible with the established multistage description of methylcellulose thermogelation, in which changes in polymer hydration and chain dynamics precede hydrophobic association, fibril formation, and development of a mechanically connected network [12,13,15,26,27]. Within this interpretation, the temporary decrease in both moduli may reflect disruption or reorganization of hydrated polymer structures before sufficient intermolecular connectivity develops to produce an elastic response. The subsequent increase in G′ is consistent with progressive association and formation of load-bearing intermolecular junctions or fibrillar structures [26].

Direct concentration-matched studies using complete heating–cooling oscillatory rheology for pure methylcellulose at 0.1 wt.% appear to be limited. Hatakeyama et al. investigated unmodified and chemically cross-linked methylcellulose over a concentration range of 0.1–4.0 wt.% and identified clear-sol-to-turbid-sol, sol-to-gel, and phase-separation transitions during heating [39]. However, that study used thermal, macroscopic, and structural criteria rather than a directly comparable G′/G″ crossover under the present oscillatory protocol.

During cooling, both G′ and G″ increased sharply and remained elevated down to 4 °C (Figure 1d). Consistently, tan(δ) remained below 1 throughout the entire cooling step, indicating no complete rheological gel–sol transition. This response indicates persistence of mechanically connected or kinetically constrained structures after formation of the high-temperature gel. The separation between association during heating and dissociation during cooling is qualitatively consistent with the pronounced thermal hysteresis previously reported for more concentrated aqueous methylcellulose systems [10,29]. Structural studies have also shown that methylcellulose fibrillar organization can persist during cooling and contribute to delayed rheological recovery [13].

Nevertheless, the absence of a reverse crossover does not demonstrate that all molecular associations remained intact. Partial chain rehydration, fibril dissociation, loss of larger scattering domains, or local mobility recovery could occur without causing G″ to exceed G′ at the selected angular frequency. In addition, mechanical loading is known to influence methylcellulose gelation and the resulting network structure and mechanical properties [25]. The continuous oscillatory deformation applied during cooling may therefore have promoted structural rearrangement or maintained contacts within the weak network, contributing to the persistent gel-like rheological response.

Although the G′/G″ crossover near 57 °C provides a useful operational heating marker, this criterion alone is insufficient to identify a unique critical gel point in the present system because it coincides with the sharp decrease and subsequent recovery of both moduli. A more rigorous determination of critical gelation would require measurements over multiple frequencies and assessment of frequency-independent tan(δ) or corresponding power law behavior according to the Winter–Chambon criterion [28]. Accordingly, the rheological transition is best described using the combined evolution of tan(δ), G′, G″, and heating–cooling hysteresis rather than relying exclusively on the condition G′ = G″. The reported value of approximately 57 °C should therefore be interpreted as a method-specific transition marker under the experimental conditions used here.

### 4.2. Kinetics of Methylcellulose Association and Dissociation

**Figure 2:**
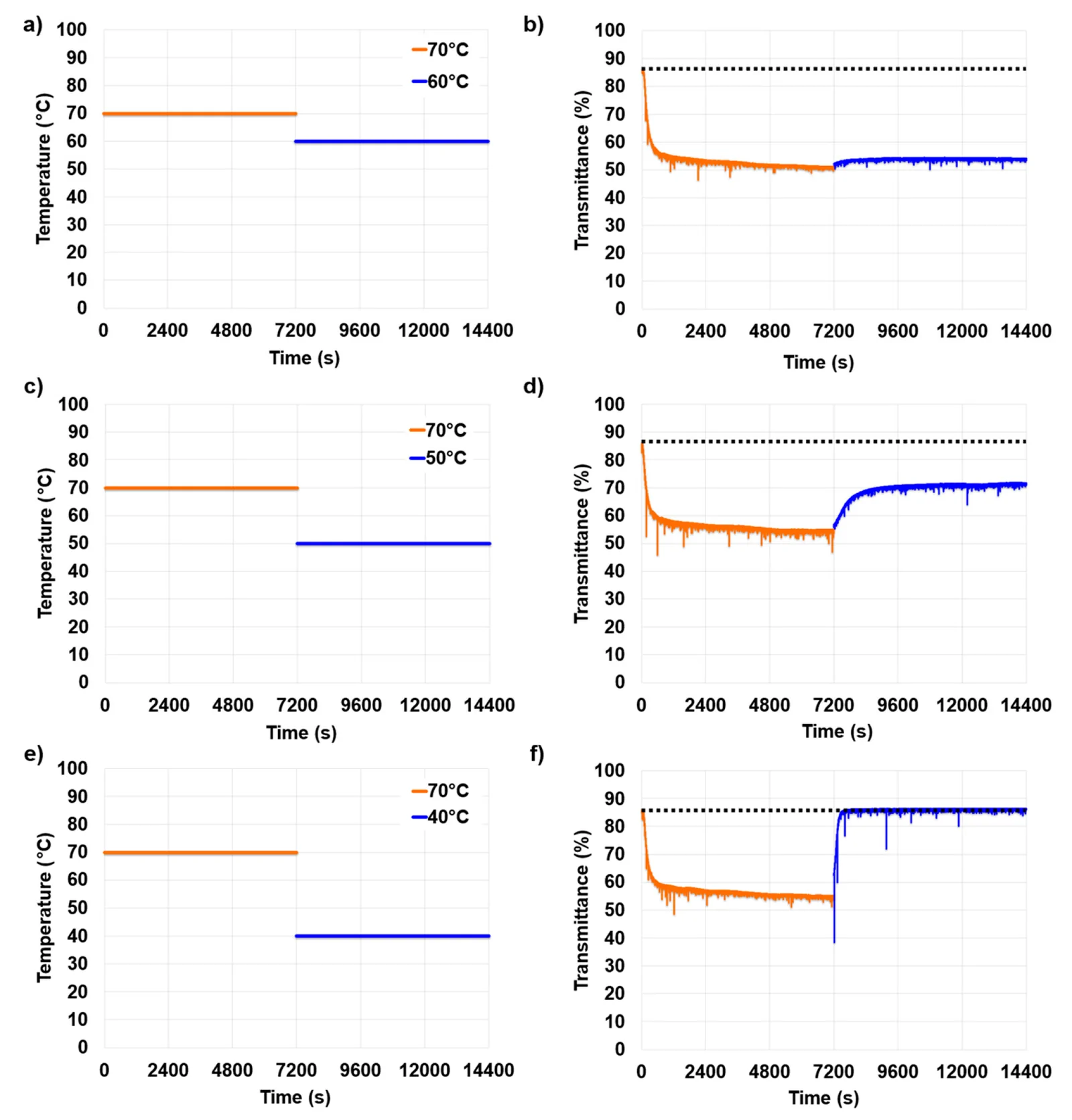
Transmittance response of the investigated 0.1 wt.% methylcellulose during temperature-jump experiments monitored using a DLS system. (**a**,**b**) 70 °C to 60 °C; (**c**,**d**) 70 °C to 50 °C; (**e**,**f**) 70 °C to 40 °C. The decrease in transmittance at 70 °C indicates the development of turbidity associated with thermally induced methylcellulose association and gel formation. Upon cooling, the recovery of transmittance depends strongly on the final temperature: limited recovery is observed at 60 °C, partial recovery at 50 °C, and nearly complete recovery at 40 °C, indicating a temperature-dependent gel-to-sol transition with pronounced hysteresis.

Transmittance measurements were used to follow time-dependent optical changes associated with methylcellulose association, aggregation, and gel formation. During the 70 °C isothermal step, the investigated 0.1 wt.% solution rapidly became turbid in all temperature-jump experiments (Figure 2b,d,f). In all cases, the transmittance decreased sharply from the initial value of approximately 85–86% to about 55–60%, followed by a slower decay toward a quasi-plateau. A similar separation between rapid initial structural development and slower aging has been reported in isothermal rheological and light scattering studies of methylcellulose, although at higher polymer concentrations [20,40]. Thus, the two-stage optical response observed here is consistent with rapid formation of scattering structures followed by slower reorganization or growth. However, because transmittance depends on the size, number density, and refractive index contrast of these structures, the slower decrease cannot be assigned uniquely to continued gel formation and may also reflect coarsening or densification of structures formed during the initial stage.

Following the temperature jumps, optical recovery depended strongly on the final temperature. Cooling to 60 °C produced only minor recovery, cooling to 50 °C produced partial recovery to approximately 70%, and cooling to 40 °C resulted in nearly complete recovery toward the initial baseline. Previous transmittance studies likewise showed that optical clarification during cooling occurs at temperatures substantially below the clouding transition observed during heating [23]. Structural investigations have also demonstrated that thermally formed methylcellulose fibrils can persist during cooling, providing a mechanistic basis for delayed recovery and thermal hysteresis [13]. Nevertheless, near-complete transmittance recovery at 40 °C indicates disappearance or weakening of the structures responsible for strong optical scattering, not necessarily complete dissociation of all molecular associations or mechanically active junctions.

The difference between the transmittance and rheological responses reflects both their distinct structural sensitivities and their different measurement protocols. Rheology detects structures capable of supporting mechanical stress, whereas transmittance responds to scattering domains regardless of whether they form a mechanically connected network. Optical clarification may therefore occur before complete mechanical relaxation. In addition, the transmittance experiments involved quiescent temperature jumps, whereas rheological cooling was performed under continuous oscillatory deformation. Overall, the results demonstrate that methylcellulose association and dissociation are both temperature- and path-dependent and that transmittance and rheology provide complementary but non-equivalent measures of structural recovery.

### 4.3. Monitoring Methylcellulose Association and Dissociation by DLS

DLS was used to further investigate the thermal response of the investigated 0.1 wt.% methylcellulose solution during continuous heating and cooling. Although commonly used for particle sizing, DLS also provides dynamic information during polymer association and gelation because the autocorrelation function reflects changes in the mobility of polymer-rich domains, aggregates, concentration fluctuations, and network-like structures. The apparent diffusion coefficient was therefore interpreted as a mobility-sensitive descriptor of the evolving methylcellulose structure [6,34,35].

**Figure 3:**
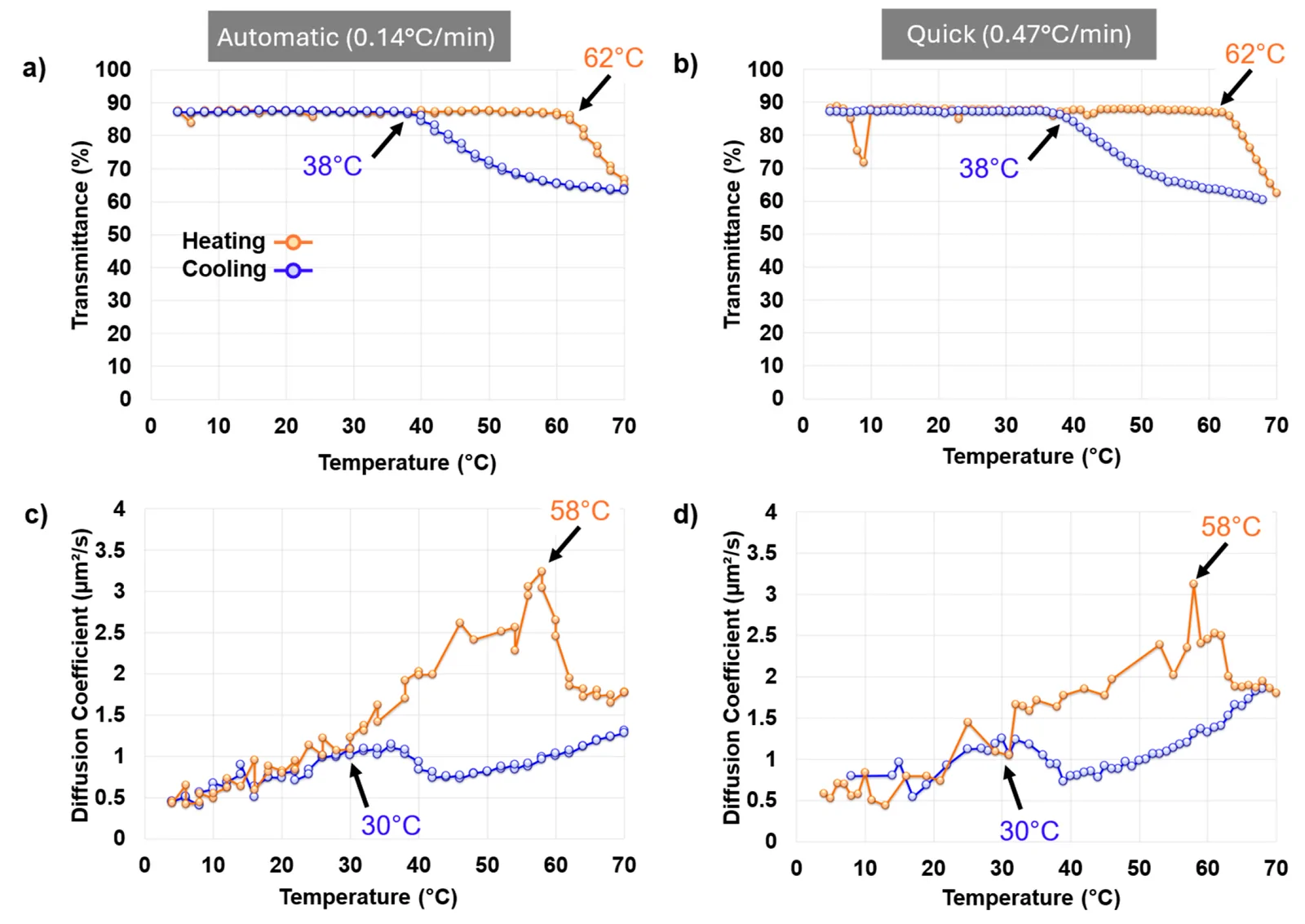
Temperature-dependent transmittance and apparent diffusion coefficient of the investigated 0.1 wt.% methylcellulose solution measured by DLS during heating and cooling. Transmittance (**a**,**b**) and apparent diffusion coefficient (**c**,**d**) were recorded using the automatic (**a**,**c**; 0.14 °C min^−1^) and quick (**b**,**d**; 0.47 °C min^−1^) acquisition modes. Both protocols show pronounced hysteresis, with the optical transition occurring near 62 °C during heating and transmittance recovery near 38 °C during cooling, while the apparent diffusion coefficient reveals earlier changes in polymer mobility and delayed recovery upon cooling.

Two DLS acquisition modes were compared: automatic mode at 0.14 °C min^−1^ and quick mode at 0.47 °C min^−1^. Both protocols showed the same qualitative behavior, with pronounced hysteresis in both transmittance and apparent diffusion coefficient (Figure 3). During heating, transmittance remained near its high-transmission plateau until approximately 62 °C and then decreased sharply, indicating formation of light scattering-associated structures (Figure 3a,b). The similarity between acquisition modes suggests that the investigated heating rate range had only minor influence on the optical transition temperature.

The apparent diffusion coefficient was more sensitive to the early stages of structural development. In the low temperature sol state, the DLS-derived apparent mobility increased gradually with temperature, consistent with enhanced Brownian mobility and the decrease in solvent viscosity upon heating (Figure 3c,d). However, a clear deviation occurred at approximately 58 °C, before the sharp decrease in transmittance observed near 62 °C. This indicates that polymer mobility became restricted before large or dense scattering structures caused significant turbidity.

During cooling, transmittance recovered near 38 °C, whereas the apparent diffusion coefficient overlapped with the heating path only at lower temperatures, around 30–32 °C. Optical clarification therefore preceded full mobility recovery. This distinction indicates that optically clear samples can still retain dynamic signatures of residual associations, weak junctions, or slowly relaxing polymer-rich regions.

The technique-dependent operational transition markers obtained for the investigated 0.1 wt.% methylcellulose formulation are summarized in Table 1. During heating, the DLS mobility marker occurred approximately 1 °C above the rheological crossover and approximately 4 °C below the sharp transmittance decrease. During cooling, optical recovery occurred approximately 6–8 °C before the DLS-derived apparent mobility returned to the heating path values, whereas no reverse rheological transition was observed within the investigated temperature range.

The differences summarized in Table 1 should not be interpreted as disagreement among the techniques, because each method probes a different aspect of the evolving structure and was applied under a distinct measurement protocol. During rheological cooling, the weak methylcellulose network is continuously subjected to oscillatory deformation, which may promote structural rearrangement, maintain contacts between polymer-rich domains, or delay relaxation of mechanically connected structures. In contrast, DLS probes the sample optically and does not impose comparable mechanical deformation. Therefore, the absence of a rheological gel–sol transition may reflect both intrinsic thermal hysteresis and the persistence of mechanically connected structures under oscillatory probing. This interpretation is consistent with previous reports that methylcellulose association and dissociation occur over different temperature ranges [10].

Overall, these results show that gelation and dissolution are not defined by a single transition temperature. Instead, the transition involves optical, dynamic, and mechanical changes that occur over different temperature ranges and depend on thermal history and measurement conditions. The influence of thermal ramp rate was not investigated systematically. Rheology was performed at 1 °C min^−1^, whereas the DLS comparison covered only the limited range of 0.14–0.47 °C min^−1^. The reported transition markers and hysteresis windows should therefore be regarded as protocol-specific, and measurements over a broader range of heating and cooling rates are required to distinguish kinetic effects from formulation effects.

### 4.4. Describing the Gelation Behavior with a Two-State Phenomenological Model

To evaluate whether the DLS-derived mobility could be described by temperature-dependent diffusion alone, the heating cycle (4–70 °C) was first fitted using a simple Arrhenius-type relationship:

(10) $$D(T)=D_{0}{exp}^{\left(-\frac{E_{a}}{RT}\right)}$$

**Figure 4:**
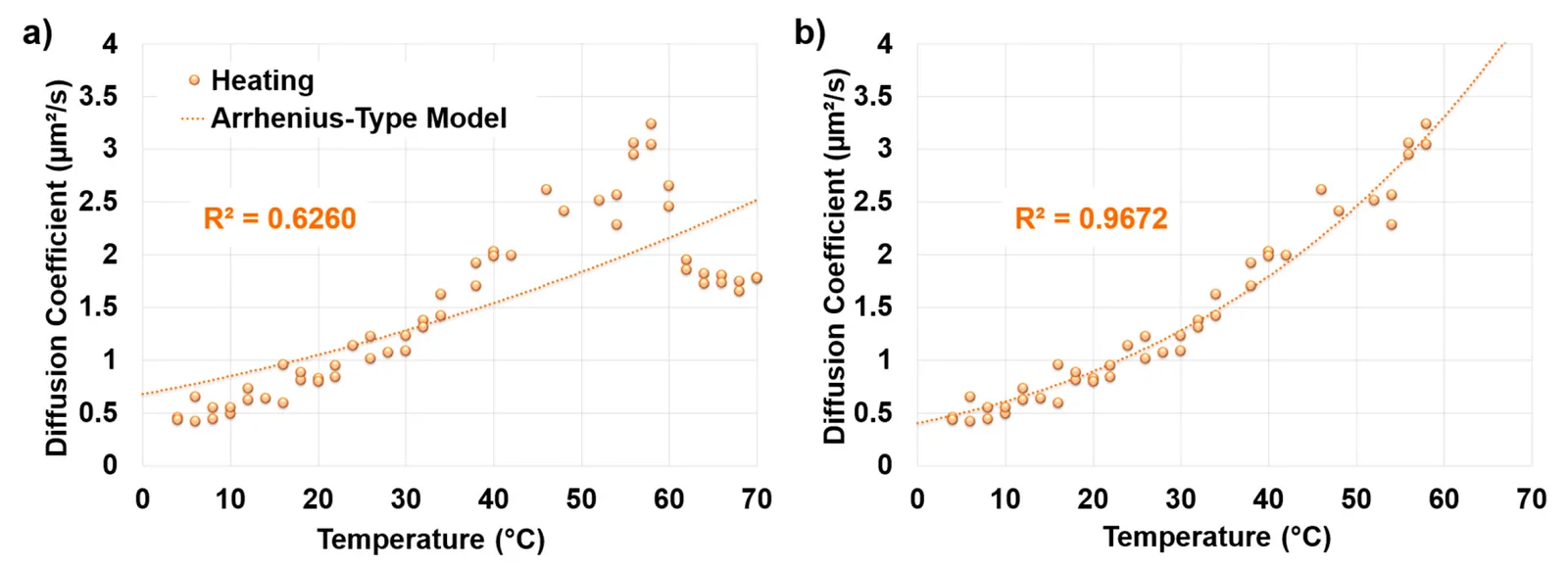
Arrhenius-type analysis of the DLS-derived apparent diffusion coefficient during heating of the investigated 0.1 wt.% methylcellulose solution. (**a**) Fit of the apparent diffusion coefficient over the full heating range using a single Arrhenius-type relationship. (**b**) Arrhenius-type fit restricted to the sol-phase region, indicating that the pre-gelation mobility of methylcellulose follows an Arrhenius-like temperature dependence.

When applied to the full heating range, this model gave a poor fit, with *R*^2^ = 0.6260 (Figure 4a), because the onset of methylcellulose association introduces a change in dynamic state. In contrast, when restricted to low-temperature sol-like regions, the model described the data well, with *R*^2^ = 0.9672 (Figure 4b), indicating that a single Arrhenius-type baseline adequately represents the apparent mobility before the main association transition. The subsequent deviation from this baseline is consistent with the onset of methylcellulose association and the emergence of additional dynamic processes. This behavior is consistent with previous DLS studies of methylcellulose, which have identified both a cooperative diffusion mode associated with concentration fluctuations in the polymer solution and a slower mode attributed to the dynamics of pre-gel clusters [6].

**Figure 5:**
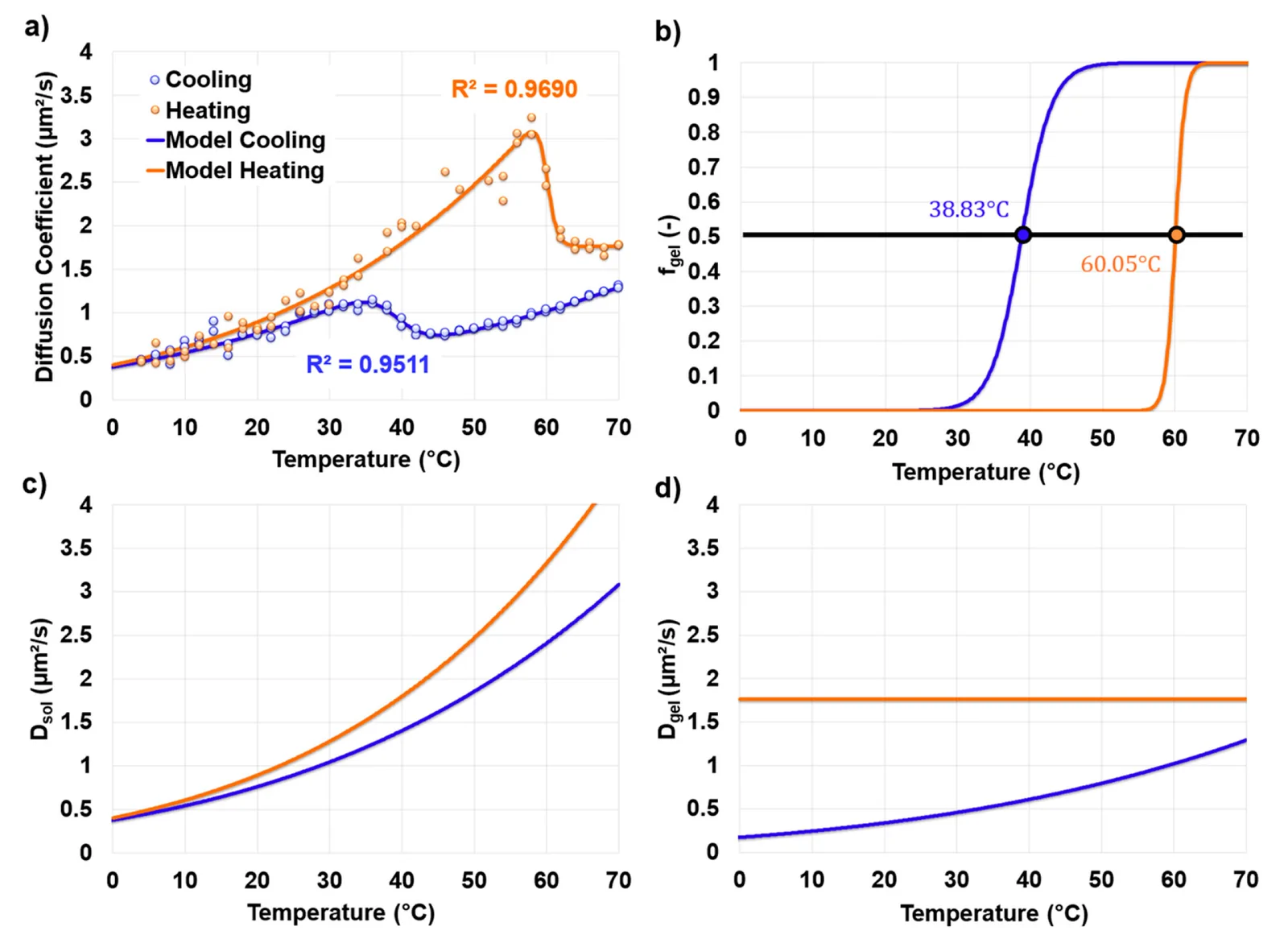
Two-state phenomenological description of methylcellulose association and dissociation in the investigated formulation based on DLS-derived mobility. (**a**) Experimental and modeled apparent diffusion coefficients of 0.1 wt.% methylcellulose during heating and cooling. (**b**) Estimated apparent gel-like fraction during heating and cooling. (**c**,**d**) Model-estimated independent apparent diffusion coefficients of the sol-like and gel-like states, respectively, during heating and cooling.

The complete heating–cooling response was subsequently described using the two-state phenomenological model. The model reproduced the apparent mobility during heating and cooling with *R*^2^ = 0.9690 and *R*^2^ = 0.9511, respectively (Figure 5a). The fitted transition midpoint was 60.05 °C during heating and 38.83 °C during cooling, corresponding to a hysteresis of approximately 21.2 °C (Figure 5b; Table 2). The transition midpoint represents the temperature at which sol-like and gel-like dynamic contributions are equal, not a strict thermodynamic phase boundary. The large separation between the heating and cooling midpoints is qualitatively consistent with previous optical, structural, NMR, and light scattering studies showing pronounced methylcellulose hysteresis, persistence of fibrillar structures during cooling, and slow reorganization of the gel network [13,23,27,40].

The transition width, ∆T, also differed markedly between heating and cooling. During heating, ∆T was 0.68 °C, indicating a sharp mobility decrease once gelation was triggered. During cooling, ∆T increased to 2.1 °C, indicating broader and smoother recovery. The broader cooling transition is consistent with a more distributed recovery process in which rehydration, dissociation, and relaxation of persistent structures occur over different time scales [13,27,40].

The fitted Arrhenius parameters further distinguish the two mobility regimes (Table 2). The sol-phase activation energies were 26.73 and 23.41 kJ mol^−1^ during heating and cooling, respectively. The gel-phase activation energy during heating ${E_{a}}_{gel}$ was fitted as 0 kJ mol^−1^, indicating an approximately temperature-independent apparent mobility of the formed gel state over the measured range. This reflects a plateau of the apparent diffusion coefficient after gelation and suggests that DLS relaxation becomes dominated by restricted network mobility rather than sol-like Brownian motion.

The model also estimates the apparent gel-like fraction and separate sol and gel state mobilities (Figure 5c,d). The cooling fit retained lower effective mobility than the heating fit, consistent with residual dynamic constraints, partially dissociated structures, or slowly relaxing network fragments. This interpretation agrees qualitatively with structural and dynamic evidence that methylcellulose can retain fibrillar structures during cooling and undergo slow microscopic reorganization after gel formation [13,27,40]. The two-state model should therefore be viewed as a simplified description of the different dynamic environments present in the system. In reality, these environments may span a broad range of mobilities rather than consist of only two distinct states. For example, tracer transport measurements in methylcellulose gels have been successfully described using either two relaxation processes or a continuous stretched exponential model [41]. Therefore, a good two-state fit does not necessarily mean that two separate thermodynamic phases are present. Instead, the calculated gel-like fraction represents the relative contribution of slower, gel-like dynamics, rather than an actual mass or phase fraction. Similarly, the sol-like and gel-like mobility values are parameters obtained from the model and should not be interpreted as independently measured diffusion coefficients.

**Figure 6:**
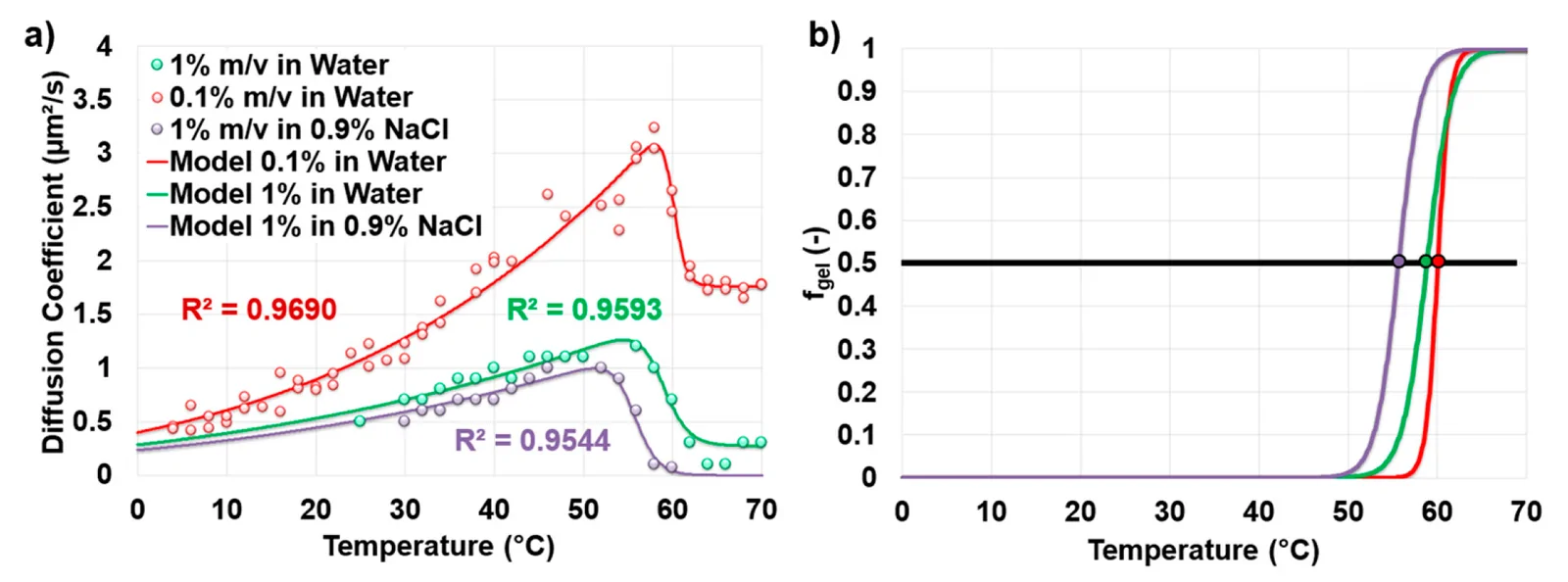
Effect of methylcellulose concentration and ionic strength on DLS-derived gelation behavior during heating. (**a**) Experimental apparent diffusion coefficients and (**b**) model-estimated apparent gel-like fraction corresponding to two-state phenomenological model fits for the investigated samples: 0.1 wt.% methylcellulose in water, 1.0 wt.% methylcellulose in water, and 1.0 wt.% methylcellulose in 0.9 wt.% NaCl.

The same model was applied to heating data for 0.1 wt.% methylcellulose in water, 1.0 wt.% methylcellulose in water, and 1.0 wt.% methylcellulose in 0.9 wt.% NaCl. The model provided good fits, with *R*^2^ values of 0.9690, 0.9593, and 0.9544, respectively (Figure 6a), showing that the two-state description can compare DLS-derived mobility across the tested formulations.

Increasing methylcellulose concentration shifted the fitted transition midpoint from 60.05 °C for 0.1 wt.% in water to 58.83 °C for 1.0 wt.% in water (Figure 6b; Table 2). This modest decrease is consistent with increased intermolecular contact and easier network formation at higher polymer concentration. The addition of 0.9 wt.% NaCl further decreased the transition midpoint to 55.64 °C for 1.0 wt.% methylcellulose, consistent with salt-assisted dehydration and hydrophobic association [42,43].

The transition width ∆T increased from 0.68 °C for 0.1 wt.% methylcellulose to 1.39 °C for 1.0 wt.% methylcellulose in water and 1.28 °C for 1.0 wt.% methylcellulose in 0.9 wt.% NaCl. Thus, the higher-concentration formulations showed broader mobility transitions, possibly reflecting more heterogeneous association. For 1.0 wt.% methylcellulose in 0.9 wt.% NaCl, the fitted gel state mobility approached zero, consistent with a strongly constrained network. The DLS-derived apparent diffusion coefficient should therefore be interpreted as a mobility descriptor of the gel network rather than simple molecular diffusion.

### 4.5. Extension of the Mobility Model to Concentration and Ionic Strength Effects

After fitting the two-state model separately to the individual datasets, the framework was extended to a composition-dependent two-state model including methylcellulose concentration, ionic strength, and thermal history. In this model, the apparent diffusion coefficient remains a weighted contribution of sol-like and gel-like states, but transition temperature and state mobilities vary with formulation composition.

**Figure 7:**
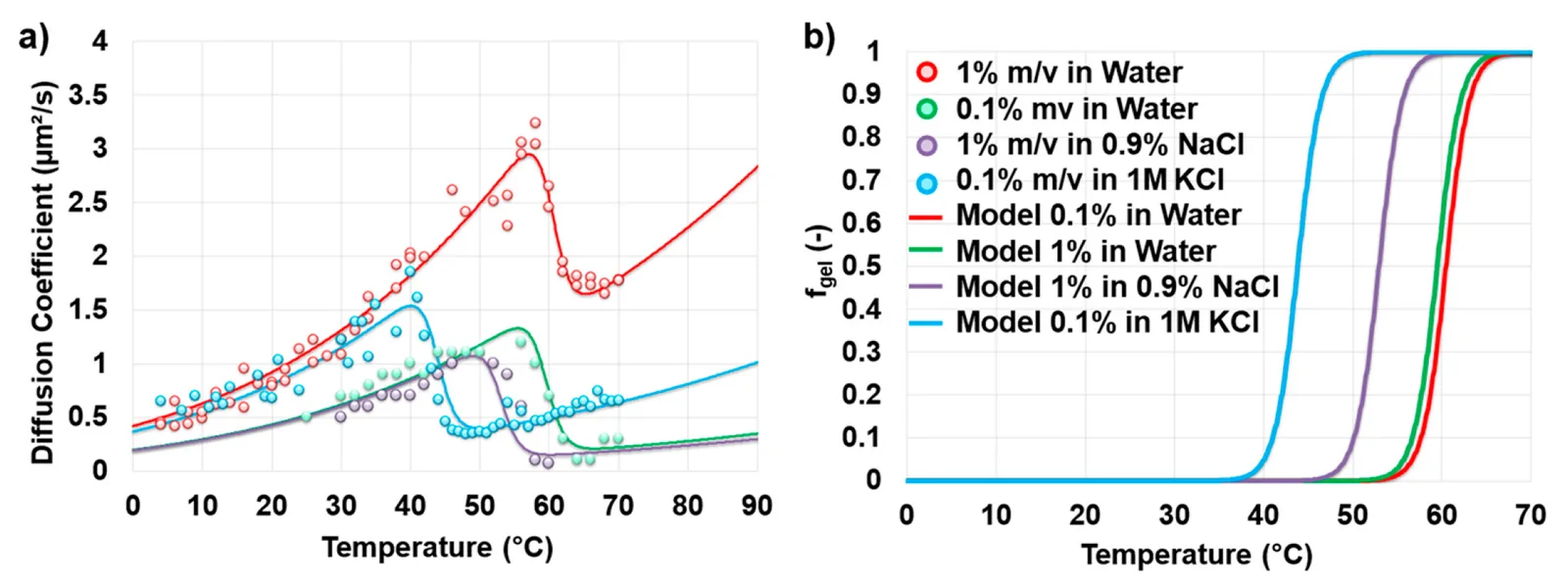
Composition-dependent two-state model for methylcellulose gelation during heating. (**a**) Experimental and modeled apparent diffusion coefficients for methylcellulose solutions with different polymer concentrations and ionic strengths. (**b**) Corresponding model-estimated apparent gel-like fraction, $f_{gel}$, showing the shift of the sol–gel transition with concentration and salt addition.

The model was calibrated simultaneously using four formulation conditions: 0.1 wt.% methylcellulose in water, 1.0 wt.% methylcellulose in water, 1.0 wt.% methylcellulose in 0.9 wt.% NaCl, and 0.1 wt.% methylcellulose in 1 M KCl (Figure 7). Using one parameter set, the model captured the main features of the measured DLS-derived apparent diffusion curves, including the pre-gelation mobility increase, the mobility decrease during gelation, and lower post-gel mobility.

The apparent gel-like fraction curves show that increasing methylcellulose concentration from 0.1 to 1.0 wt.% shifted the transition slightly to lower temperature (Figure 7b). Salt addition produced a stronger shift: 1.0 wt.% methylcellulose in 0.9 wt.% NaCl gelled at lower temperature than the corresponding 1.0 wt.% solution in water, and 0.1 wt.% methylcellulose in 1 M KCl showed a marked decrease in estimated transition temperature.

**Figure 8:**
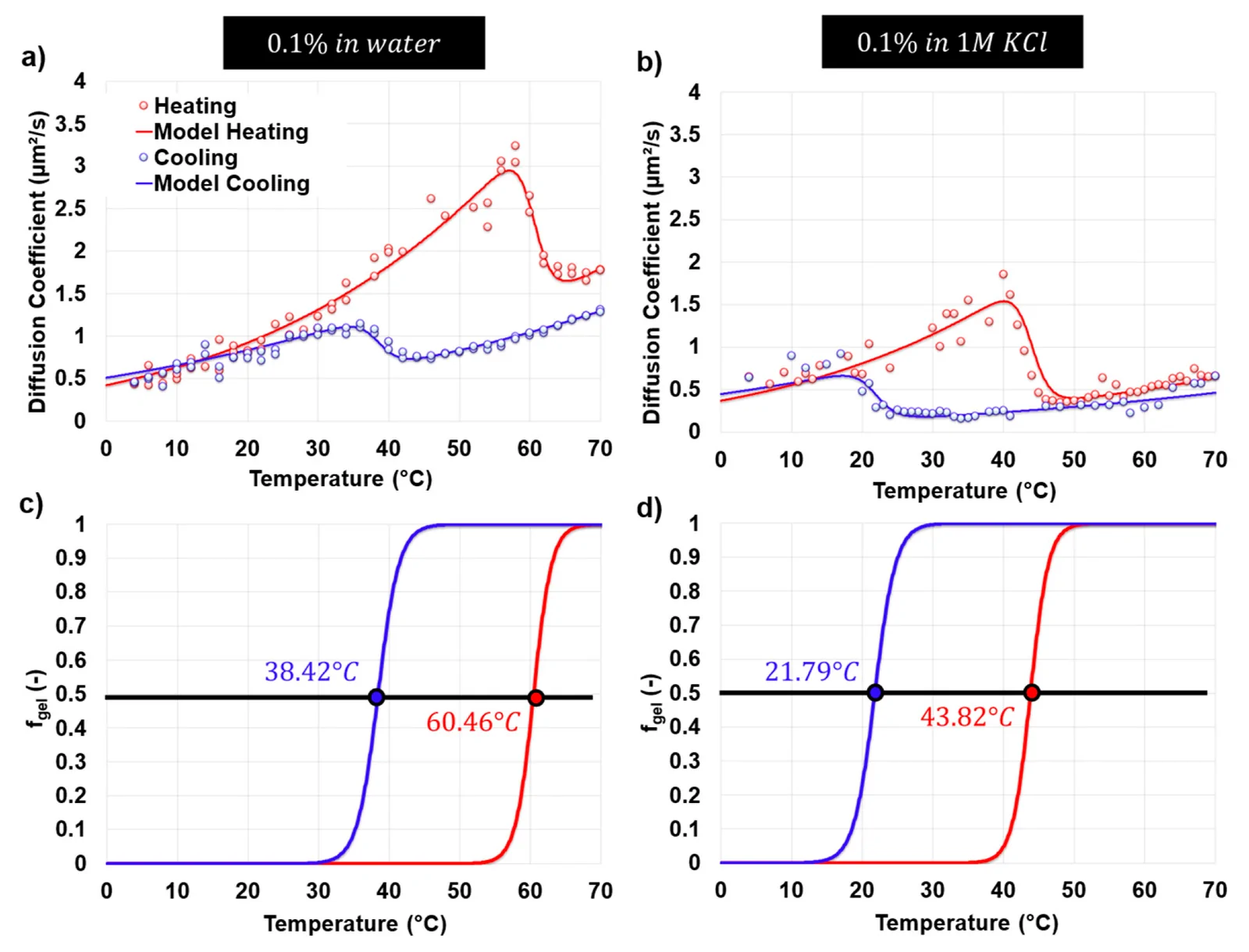
Composition-dependent two-state model calibration of heating–cooling hysteresis in the investigated 0.1 wt.% methylcellulose. Experimental and modeled apparent diffusion coefficients during heating and cooling for 0.1 wt.% methylcellulose in water (**a**) and 0.1 wt.% methylcellulose in 1 M KCl (**b**). The corresponding model-estimated apparent gel-like fraction profiles are shown in (**c**) and (**d**), respectively. The transition midpoint, defined as *f_gel_* = 0.5, shifts from 60.46 °C during heating and 38.42 °C during cooling in water to 43.82 °C and 21.79 °C in 1 M KCl, demonstrating the strong effect of ionic strength on both sol–gel and gel–sol transitions.

Heating and cooling cycles were compared for 0.1 wt.% methylcellulose in water and 0.1 wt.% methylcellulose in 1 M KCl (Figure 8). For the salt-free formulation, transition midpoints were approximately 60.46 °C during heating and 38.42 °C during cooling. In 1 M KCl, they shifted to approximately 43.82 °C and 21.79 °C, respectively. Thus, salt addition lowered both sol–gel and gel–sol transition temperatures while preserving pronounced hysteresis.

However, the model treats salt effects through an ionic strength-dependent term and does not explicitly distinguish salt identity. This is an important limitation, because MC gelation can be ion-specific, with salting-out salts generally lowering gelation temperature and salting-in salts shifting it upward [24,29]. Because NaCl and KCl were not tested at matched polymer concentrations and molar salt concentrations, the present dataset cannot fully separate ionic strength effects from ion-specific effects.

The fitted parameters of the composition-dependent two-state model are summarized in Table 3. In this global formulation, the concentration- and ionic strength-dependent terms (a, b, υ, $\alpha_{sol}$, $\alpha_{gel}$, $\beta_{sol}$, and $\beta_{gel}$) are shared across the fitted datasets, whereas thermal history effects are represented through separate heating and cooling transition and mobility parameters.

The global calibration represents a compromise rather than independently optimized fits for each condition. The model gave overall *R*^2^ values of 0.8376 for heating and 0.8188 for cooling (Table 4). The best fits were obtained for 0.1 wt.% and 1.0 wt.% methylcellulose in water, whereas lower *R*^2^ values were obtained for 0.1 wt.% methylcellulose in 1 M KCl and especially 1.0 wt.% methylcellulose in 0.9 wt.% NaCl. The latter likely reflects higher experimental scatter and fewer reliable low temperature data points, which weakens constraint of the sol state mobility baseline.

Overall, the composition-dependent model captures the main trends in concentration, salt, and thermal history dependence, but should be interpreted as a calibrated phenomenological interpolation framework rather than a universally predictive model.

### 4.6. Model-Based Estimations Across the Formulation Space

**Figure 9:**
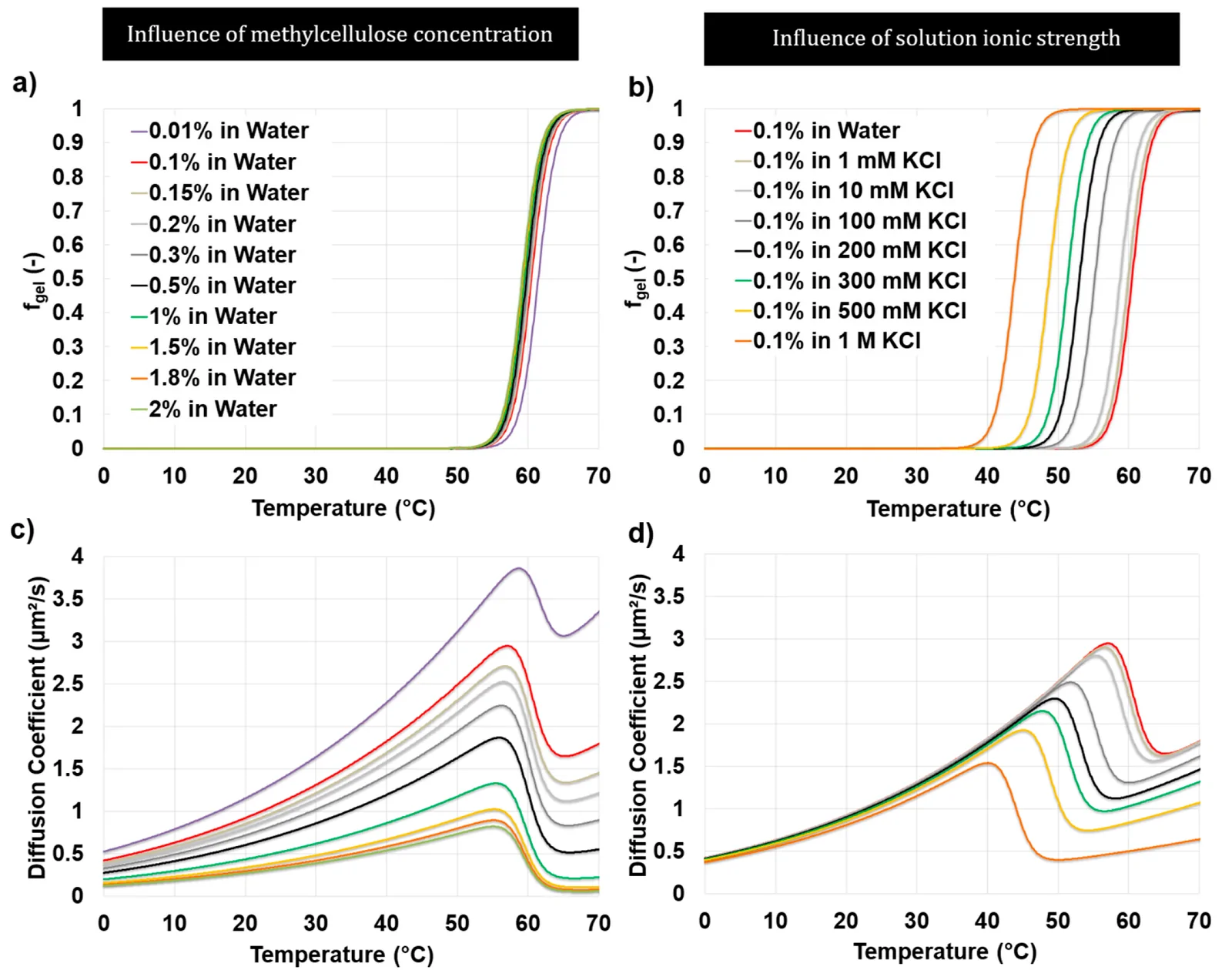
Model-based estimates of the effects of methylcellulose concentration and ionic strength on gelation during heating. Model-estimated apparent gel-like fraction (**a**,**b**) and DLS-derived apparent mobility (**c**,**d**) as functions of temperature. Panels (**a**,**c**) show the modeled effect of methylcellulose concentration in water, whereas panels (**b**,**d**) show the modeled effect of KCl concentration for 0.1 wt.% methylcellulose. Intermediate conditions represent model interpolation, whereas conditions outside the calibrated ranges represent extrapolation; neither was independently validated.

After calibration, the composition-dependent model was used to visualize how methylcellulose concentration and KCl concentration affect DLS-derived apparent mobility and apparent gel-like fraction during heating (Figure 9). The calculations include both interpolations between experimentally investigated formulation levels and extrapolations beyond the calibrated formulation range. Because the estimates were not tested against independent measurements at the corresponding intermediate or out-of-range conditions, they should be interpreted as unvalidated model-based estimates, with particular caution applied to the extrapolations.

Within the modeled range, increasing methylcellulose concentration decreased the estimated apparent mobility, especially after gelation, consistent with denser and more constrained polymer networks. The model also estimated a slight decrease in transition temperature with increasing polymer concentration, reflecting increased probability of intermolecular association [11,26]. Within the simulated range, however, the concentration-induced transition shift was modest. Because concentration and ionic strength effects are represented through predefined power law and linear terms, respectively, the smooth monotonic trends in Figure 9 partly reflect the selected model structure and should not be interpreted as independently established mechanistic laws.

The influence of KCl concentration was more pronounced. Increasing KCl shifted the apparent gel-like fraction curves to lower temperatures and reduced post-gel mobility, consistent with salt-assisted dehydration, enhanced hydrophobic association, and formation of a more constrained network [9,29,42]. The model therefore suggests that methylcellulose concentration primarily affects network mobility and post-gel restriction, whereas ionic strength has a stronger influence on transition temperature. Because NaCl and KCl were not tested at matched polymer and salt concentrations, the KCl curves should not be interpreted as validated ion-specific predictions.

These model-based estimates require experimental verification. Nevertheless, they illustrate how the calibrated framework can be used to compare formulation trends and identify potential processing windows in which methylcellulose solutions remain mobile, begin to associate, or form dynamically restricted gels.

### 4.7. Comparison with Independent Literature Trends

**Figure 10:**
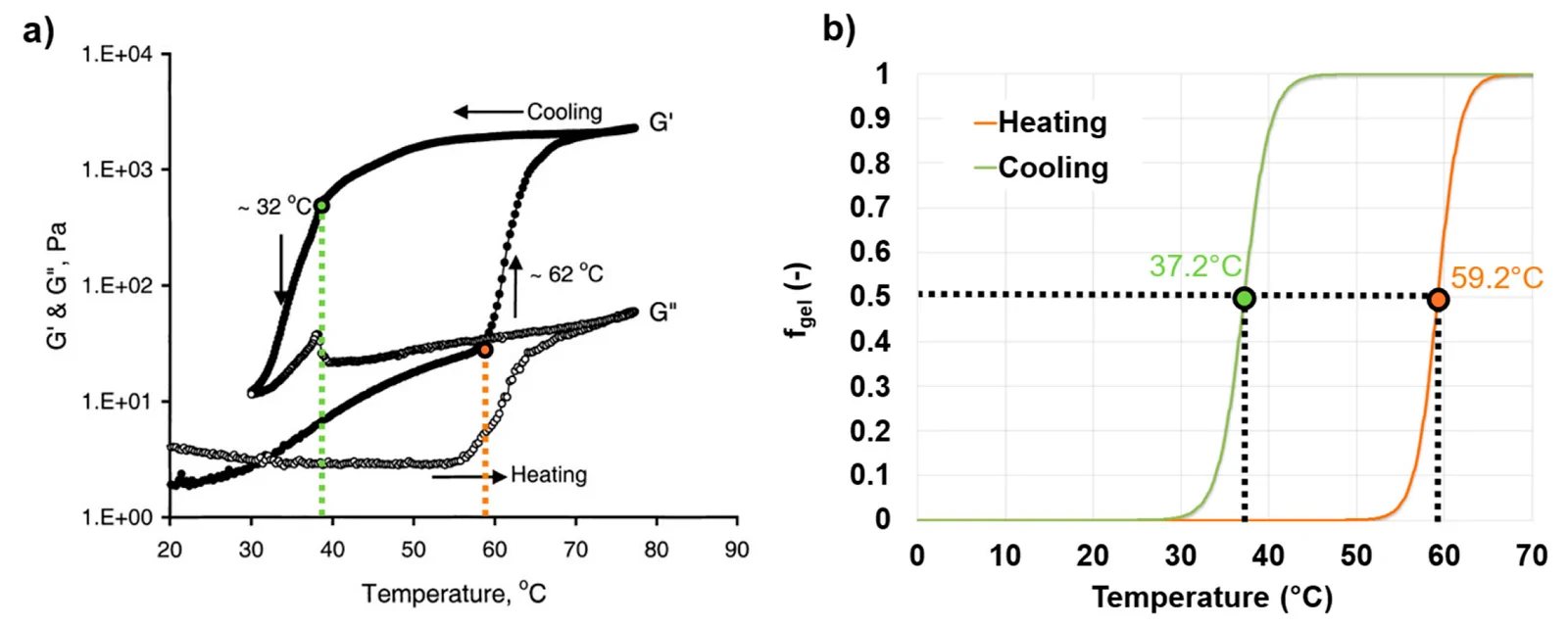
Qualitative comparison of literature rheological gelation markers with the model-estimated apparent gel-like fraction. (**a**) Literature oscillatory rheology data for a 1.8 wt.% SM4000 methylcellulose solution during heating and cooling at 1 rad s^−1^ and approximately 1 °C min^−1^, adapted from Li, Wang, and Xu [29]. (**b**) Composition-dependent two-state model estimation of the apparent gel-like fraction during heating and cooling. The estimated transition midpoints are close to the temperatures associated with the abrupt increase and decrease of G′, supporting the use of $f_{gel}$ = 0.5 as an effective gelation/degelation marker.

The external consistency of the composition-dependent model was assessed by comparison with independent literature rheology and turbidity data. Li, Wang, and Xu reported thermoreversible gelation of a 1.8 wt.% SM4000 methylcellulose solution using oscillatory rheology at 1 rad s^−1^ and approximately 1 °C min^−1^ [29]. In their measurements, G′ increased sharply near 62 °C during heating and decreased sharply near 32 °C during cooling (Figure 10a). Because a clear G′ = G″ crossover criterion was not observed, the abrupt G′ changes, supported by DSC, provide more meaningful transition markers than a simple crossover criterion.

The model-estimated apparent gel-like fraction showed similar transition behavior (Figure 10b). The estimated midpoint occurred at approximately 59.2 °C during heating and 37.2 °C during cooling, close to the temperature range of the rheological transitions. Although the model was calibrated using DLS-derived mobility rather than rheological moduli, this qualitative agreement supports the interpretation of the apparent gel-like fraction as a useful mobility-based gelation marker.

**Figure 11:**
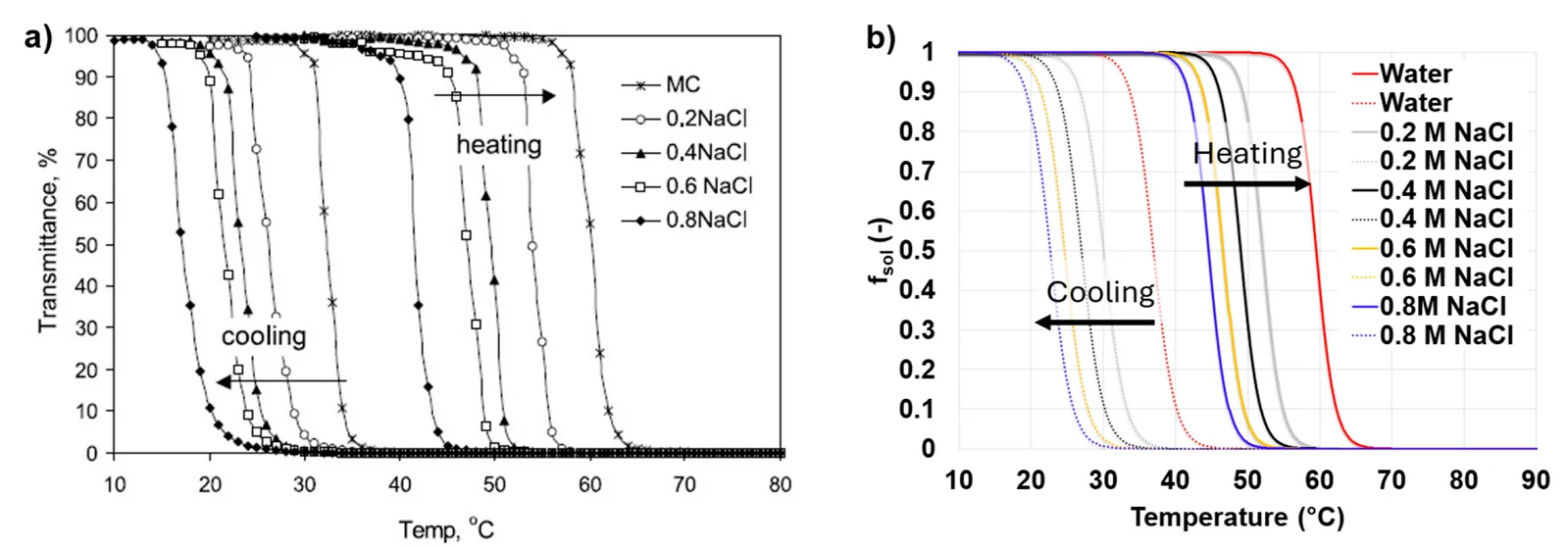
Qualitative comparison of literature turbidity measurements with the model-estimated sol-like fraction. (**a**) Literature transmittance curves of methylcellulose solutions during heating and cooling at different NaCl concentrations; adapted from Xu and Li [23]. (**b**) Model-estimated sol fraction, $f_{sol}$ = 1 − $f_{gel}$, for corresponding ionic strength conditions. Although transmittance is not explicitly modeled in the composition-dependent two-state framework, the estimated sol fraction reproduces the main qualitative trends observed experimentally, including thermal hysteresis and the salt-induced shift of the transition to lower temperatures.

In Figure 11, the model was also compared with turbidity data reported by Xu and Li, who showed that methylcellulose transmittance decreases upon heating and recovers upon cooling, with NaCl shifting the transition to lower temperature [23]. Although transmittance is not included explicitly in the model, the estimated sol-like fraction $f_{sol}$ reproduced the main qualitative features of the turbidity curves: loss of sol-like character during heating, recovery during cooling at lower temperature, thermal hysteresis, and salt-induced shifts in the transition temperature.

Because the formulations and measurement protocols do not exactly match those used for model calibration, the literature comparisons should be regarded as qualitative external consistency checks rather than formal quantitative validation. Together, these comparisons show that the DLS-based model is consistent with established rheological and optical descriptions of methylcellulose gelation. Specifically, the apparent gel-like fraction shows qualitative correspondence with abrupt rheological changes, whereas the apparent sol-like fraction captures the principal trends in transmittance, including thermal hysteresis and salt-induced transition shifts. This consistency supports the use of DLS-derived apparent mobility as a bridge between microscopic or mesoscale dynamics and macroscopic mechanical or optical gelation markers.

## 5. Conclusions

This work developed a mobility-based framework to describe thermoresponsive association, dissociation, and hysteresis in methylcellulose systems. The DLS-derived apparent diffusion coefficient was used as a mobility-sensitive descriptor, rather than treating DLS only as a particle-sizing method. By combining rheology, transmittance, and DLS, this study showed that methylcellulose gelation cannot be described by a single transition temperature, but involves distinct mechanical, optical, and mobility-based transitions.

For the investigated 0.1 wt.% methylcellulose solution, oscillatory rheology showed a sol–gel transition during heating between approximately 50 and 60 °C, with an apparent transition near 57 °C. During cooling, tan(δ) remained below 1, and G′ stayed higher than G″, indicating persistent gel-like mechanical behavior and pronounced hysteresis. Transmittance measurements showed complementary behavior: the optically turbid state partially recovered at 50 °C and nearly fully recovered at approximately 40 °C. Thus, optical clarification occurred even though rheology indicated persistent mechanical connectivity.

DLS provided an intermediate dynamic perspective. During heating, the apparent diffusion coefficient showed mobility restriction near 58 °C before the main transmittance transition near 62 °C. During cooling, transmittance recovered near 38 °C, whereas apparent mobility recovered only around 30–32 °C. These results indicate that DLS can detect early association and delayed mobility recovery that are not fully captured by optical turbidity alone.

A simple Arrhenius-type relationship described sol state mobility but failed over the full heating range, confirming that the thermal response cannot be treated as a single diffusion process. The two-state phenomenological model addressed this by representing the measured apparent mobility as the weighted contribution of sol-like and gel-like dynamic states. For 0.1 wt.% methylcellulose, the model captured heating and cooling behavior with a hysteresis window of approximately 22 °C. The fitted transition midpoint represents the temperature at which sol-like and gel-like dynamic contributions are equal, while the transition width describes the sharpness of conversion.

The model was extended to include methylcellulose concentration and ionic strength through a composition-dependent two-state framework. Increasing polymer concentration reduced post-gel mobility and slightly lowered the fitted gelation temperature, consistent with denser and more constrained networks. Salt addition produced a stronger decrease in transition temperature and post-gel mobility, consistent with reduced hydration and enhanced hydrophobic association. The composition-dependent model yielded overall *R*^2^ values of 0.8376 for heating and 0.8188 for cooling. However, because the dataset contains a limited number of formulation conditions and does not test NaCl and KCl at matched polymer and salt concentrations, ion-specific effects cannot be separated from ionic strength effects. The model should therefore be interpreted as a calibrated phenomenological framework, not a universal predictive model.

Comparison with literature rheology and turbidity data showed that the model estimations are consistent with conventional gelation markers. The estimated apparent gel-like fraction showed qualitative correspondence with abrupt changes in G′, while the estimated sol-like fraction captured the principal qualitative trends in transmittance, including hysteresis and salt-induced transition shifts.

Overall, DLS-derived apparent mobility provides a formulation-relevant descriptor of methylcellulose gelation, dissociation, and hysteresis. The proposed framework resolves the thermal response into mobility states, transition widths, and hysteresis windows, which are directly relevant for formulation design and process optimization. With further validation across additional polymer concentrations, salt identities, salt levels, methylcellulose molar masses, substitution patterns, and polydispersities, this mobility-based approach could support rational design of thermoresponsive polysaccharide formulations for food, pharmaceutical, biomedical, coating, and extrusion-based applications.

## Figures and Tables

**Table 1:** Comparison of technique-dependent operational heating and cooling transition markers obtained by oscillatory rheology, DLS-derived apparent mobility, and transmittance for the investigated 0.1 wt.% methylcellulose formulation in water.

Method	Heating Marker	Cooling Marker	Hysteresis	Primary Response
Rheology	~57 °C	Not observed down to 4 °C	Not determined	Mechanical network formation/persistence
DLS mobility	~58 °C	~30–32 °C	~26–28 °C	Mobility restriction/recovery
Transmittance	~62 °C	~38–40 °C	~24–26 °C	Turbidity formation/optical clarification

Note: Values are approximate and correspond to method-specific operational criteria. The DLS values are direct operational markers identified from the experimental curves and are distinct from the model-fitted transition midpoints reported in Table 2. Apparent hysteresis was calculated as the difference between the corresponding heating and cooling markers. No reverse rheological transition was observed within the investigated cooling range; therefore, rheological hysteresis could not be determined.

**Table 2:** Fitted two-state model parameters describing the DLS-derived apparent diffusion behavior of the investigated methylcellulose at different concentrations and ionic strengths.

Parameter	Heating 0.1 wt.% in Water	Cooling 0.1 wt.% in Water	Heating 1.0 wt.% in Water	Heating 1.0 wt.% in 0.9 wt.% NaCl
${D_{0}}_{sol}\;$ (µm^2^/s)	51,721.35	11,280.97	2736.31	2736.31
${E_{a}}_{sol}$ (J/mol)	26,732.28	23,411.17	20,833.58	21,272.09
${D_{0}}_{gel}$ (µm^2^/s)	1.76	3284.06	0.28	0.00
${E_{a}}_{gel}$ (J/mol)	0.00	22,371.27	0.00	0.00
$T_{gel}$ (°C)	60.05	38.83	58.83	55.64
∆T (°C)	0.68	2.10	1.39	1.28

Note: Because confidence intervals and parameter covariance were not evaluated, differences among fitted activation energies, mobility pre-factors, and transition widths should be interpreted descriptively rather than as statistically resolved differences.

**Table 3:** Fitting parameters of the composition-dependent two-state model used to describe the apparent mobility behavior of the methylcellulose solutions investigated, as determined by DLS, at different polymer concentrations and ionic strengths.

Condition	Heating	Heating and Cooling ^1^	Cooling
${E_{a}}_{sol}$ (J/mol)	26,123.260		16,474.375
${D_{0}}_{sol}\;$ (µm^2^/s)	57,172.877		988.293
${E_{a}}_{gel}$ (J/mol)	23,689.706		20,420.171
${D_{0}}_{gel}$ (µm^2^/s)	17,598.097		4036.880
$T_{0}$ (°C)	60.462		38.429
a (°C)		0.429	
b (°C)		16.642	
$\alpha_{sol}$ ($L^{2.65}\cdot g^{-2.65}$)		0.319	
$\alpha_{gel}$$\;(L^{2.65}\cdot g^{-2.65}$)		0.889	
υ (-)		0.526	
∆T (°C)	1.281		1.482
$\beta_{sol}$ (L/mol)		0.130	
$\beta_{gel}$ (L/mol)		1.029	

^1^ Parameters that remain the same regardless of whether it is a heating or cooling step.

**Table 4:** Goodness-of-fit values for the composition-dependent two-state model.

Condition	*R*^2^ Heating	*R*^2^ Cooling
1.0 wt.% in water	0.9244	-
0.1 wt.% in water	0.9645	0.8839
0.1 wt.% in 1 M KCl	0.8377	0.7536
1.0 wt.% in 0.9 wt.% NaCl	0.6237	-
Overall	0.8376	0.8188

## Data Availability

The raw data supporting the conclusions of this article will be made available by the authors on request.

## Supplementary Materials

The following supporting information can be downloaded at https://www.mdpi.com/article/10.3390/gels12090766/s1, Table S1: Input measurement parameters used for particle size and shape analysis; Figure S1: Particle size and shape characterization of the methylcellulose powder by dynamic image analysis (Litesizer DIA 500, Anton Paar) using a Dry Jet dispersion unit. (**a**) Volume-based particle size distribution expressed as the equivalent diameter (xA). (**b**) Representative particle images classified according to equivalent diameter (xA) and aspect ratio, illustrating the irregular morphology of the powder.

## Abbreviations

The following abbreviations are used in this manuscript:

MC	Methylcellulose
DLS	Dynamic Light Scattering

## References

[1] Nasatto P.L., Pignon F., Silveira J.L.M., Duarte M.E.R., Noseda M.D., Rinaudo M. (2015). Methylcellulose, a cellulose derivative with original physical properties and extended applications. Polymers.

[2] Coughlin M.L., Liberman L., Ertem S.P., Edmund J., Bates F.S., Lodge T.P. (2021). Methyl cellulose solutions and gels: Fibril formation and gelation properties. Prog. Polym. Sci..

[3] Haque A., Morris E.R. (1993). Thermogelation of methylcellulose. Part I: Molecular structures and processes. Carbohydr. Polym..

[4] Chevillard C., Axelos M.A.V. (1997). Phase separation of aqueous solution of methylcellulose. Colloid Polym. Sci..

[5] Hirrien M., Chevillard C., Desbrières J., Axelos M.A.V., Rinaudo M. (1998). Thermogelation of methylcelluloses: New evidence for understanding the gelation mechanism. Polymer.

[6] Kobayashi K., Huang C.-I., Lodge T.P. (1999). Thermoreversible gelation of aqueous methylcellulose solutions. Macromolecules.

[7] Desbrières J., Hirrien M., Ross-Murphy S.B. (2000). Thermogelation of methylcellulose: Rheological considerations. Polymer.

[8] Li L., Thangamathesvaran P.M., Yue C.Y., Tam K.C., Hu X., Lam Y.C. (2001). Gel network structure of methylcellulose in water. Langmuir.

[9] Li L. (2002). Thermal gelation of methylcellulose in water: Scaling and thermoreversibility. Macromolecules.

[10] Li L., Shan H., Yue C.Y., Lam Y.C., Tam K.C., Hu X. (2002). Thermally induced association and dissociation of methylcellulose in aqueous solutions. Langmuir.

[11] Arvidson S.A., Lott J.R., McAllister J.W., Zhang J., Bates F.S., Lodge T.P., Sammler R.L., Li Y., Brackhagen M. (2013). Interplay of phase separation and thermoreversible gelation in aqueous methylcellulose solutions. Macromolecules.

[12] Lott J.R., McAllister J.W., Arvidson S.A., Bates F.S., Lodge T.P. (2013). Fibrillar structure of methylcellulose hydrogels. Biomacromolecules.

[13] Lott J.R., McAllister J.W., Wasbrough M., Sammler R.L., Bates F.S., Lodge T.P. (2013). Fibrillar structure in aqueous methylcellulose solutions and gels. Macromolecules.

[14] McAllister J.W., Schmidt P.W., Dorfman K.D., Lodge T.P., Bates F.S. (2015). Thermodynamics of aqueous methylcellulose solutions. Macromolecules.

[15] Schmidt P.W., Morozova S., Owens P.M., Adden R., Li Y., Bates F.S., Lodge T.P. (2018). Molecular weight dependence of methylcellulose fibrillar networks. Macromolecules.

[16] Schmidt P.W., Morozova S., Ertem S.P., Coughlin M.L., Davidovich I., Talmon Y., Reineke T.M., Bates F.S., Lodge T.P. (2020). Internal structure of methylcellulose fibrils. Macromolecules.

[17] Liberman L., Schmidt P.W., Coughlin M.L., Matatyaho Ya’akobi A., Davidovich I., Edmund J., Ertem S.P., Morozova S., Talmon Y., Bates F.S. (2021). Salt-dependent structure in methylcellulose fibrillar gels. Macromolecules.

[18] Liao Q., Ren H., Xu J., Wang P., Yuan B., Zhang H. (2024). Combined experiments and molecular simulations for understanding the thermo-responsive behavior and gelation of methylated glucans with different glycosidic linkages. J. Colloid Interface Sci..

[19] Kronenberger S., Jayaraman A. (2025). Effect of methylcellulose chain design on gelation and fibril structure using coarse-grained modeling. Chem. Mater..

[20] Sarkar N. (1995). Kinetics of thermal gelation of methylcellulose and hydroxypropylmethylcellulose in aqueous solutions. Carbohydr. Polym..

[21] Xu Y., Wang C., Tam K.C., Li L. (2004). Salt-assisted and salt-suppressed sol–gel transitions of methylcellulose in water. Langmuir.

[22] Xu Y., Li L., Zheng P., Lam Y.C., Hu X. (2004). Controllable gelation of methylcellulose by a salt mixture. Langmuir.

[23] Xu Y., Li L. (2005). Thermoreversible and salt-sensitive turbidity of methylcellulose in aqueous solution. Polymer.

[24] Isobe N., Shimizu S. (2020). Salt-induced LCST-type thermal gelation of methylcellulose: Quantifying non-specific interactions via fluctuation theory. Phys. Chem. Chem. Phys..

[25] Nelson A.Z., Wang Y., Wang Y., Margotta A.S., Sammler R.L., Izmitli A., Katz J.S., Curtis-Fisk J., Li Y., Ewoldt R.H. (2022). Gelation under stress: Impact of shear flow on the formation and mechanical properties of methylcellulose hydrogels. Soft Matter.

[26] Niemczyk-Soczyńska B., Sajkiewicz P., Gradys A. (2022). Toward a better understanding of the gelation mechanism of methylcellulose via systematic DSC studies. Polymers.

[27] Besghini D., Mauri M., Hashemi P., Knarr M., Adden R., Mischnick P., Simonutti R. (2023). Time-domain NMR elucidates fibril formation in methylcellulose hydrogels. Macromolecules.

[28] Winter H.H., Chambon F. (1986). Analysis of linear viscoelasticity of a crosslinking polymer at the gel point. J. Rheol..

[29] Li L., Wang Q., Xu Y. (2003). Thermoreversible association and gelation of methylcellulose in aqueous solutions. Nihon Reoroji Gakkaishi.

[30] Bodvik R., Dedinaite A., Karlson L., Bergström M., Bäverbäck P.M., Pedersen J.S., Edwards K., Karlsson G., Varga I., Claesson P.M. (2010). Aggregation and network formation of aqueous methylcellulose and hydroxypropylmethylcellulose solutions. Colloids Surf. A Physicochem. Eng. Asp..

[31] Danieli I., Rittel D. (2025). Innovative colorimetric thermal study of methylcellulose hydrogel via smartphone imaging. Sci. Rep..

[32] Joosten J.G.H., Geladé E.T.F., Pusey P.N. (1990). Dynamic light scattering by nonergodic media: Brownian particles trapped in polyacrylamide gels. Phys. Rev. A.

[33] Ikkai F., Shibayama M. (1999). Static inhomogeneities in thermoreversible physical gels. Phys. Rev. Lett..

[34] Shibayama M., Norisuye T. (2002). Gel formation analyses by dynamic light scattering. Bull. Chem. Soc. Jpn..

[35] Matsunaga T., Shibayama M. (2007). Gel point determination of gelatin hydrogels by dynamic light scattering and rheological measurements. Phys. Rev. E.

[36] Arai K., Horikawa Y., Shikata T., Iwase H. (2020). Reconsideration of the conformation of methyl cellulose and hydroxypropyl methyl cellulose ethers in aqueous solution. RSC Adv..

[37] Saiki E., Yoshida M., Kurahashi K., Iwase H., Shikata T. (2022). Elongated rodlike particle formation of methyl cellulose in aqueous solution. ACS Omega.

[38] Singh R.P. (2022). Temperature-dependent dynamic light scattering studies on dilute aqueous solution of methylcellulose. J. Polym. Res..

[39] Hatakeyama H., Onishi T., Endo T., Hatakeyama T. (2007). Gelation of chemically cross-linked methylcellulose studied by DSC and AFM. Carbohydr. Polym..

[40] Schupper N., Rabin Y., Rosenbluh M. (2008). Multiple stages in the aging of a physical polymer gel. Macromolecules.

[41] Jee A.-Y., Curtis-Fisk J.L., Granick S. (2014). Nanoparticle diffusion in methycellulose thermoreversible association polymer. Macromolecules.

[42] Alamprese C., Mariotti M. (2015). Modelling of methylcellulose thermogelation as a function of polymer concentration and dissolution media properties. LWT Food Sci. Technol..

[43] Joshi S.C., Lam Y.C. (2006). Modeling heat and degree of gelation for methyl cellulose hydrogels with NaCl additives. J. Appl. Polym. Sci..

