# Supplementary material for: DLS-Derived Apparent Mobility as a Formulation-Relevant Descriptor of Thermoresponsive Methylcellulose Gelation and Hysteresis
Source: Gels. 2026 Aug 26;12(9):766. doi: 10.3390/gels12090766 (PMC13606277; doi:10.3390/gels12090766)
Supplement: Supplementary file 1 [file gels-12-00766-s001.zip › gels-4496959-supplementary.pdf]

DLS-derived Apparent Mobility as a Formulation-Relevant Descriptor of Thermoresponsive Methylcellulose Gelation and Hysteresis

Franz Miller Branco Ferraz [1,] *, Christina Reichart [1], Laura Kainz [1] and Christian Moitzi [1]

[1] Anton Paar GmbH, Anton-Paar-Strasse 20, 8054 Graz, Austria
* Correspondence: franz.miller@anton-paar.com

The particle size and morphology of the as-received methylcellulose powder were characterized by dynamic image analysis using a Litesizer DIA 500 (Anton Paar) equipped with a Dry Jet dispersion unit. This characterization documents the physical properties of the commercial starting material used throughout the study. Although the dry-powder particle size is not directly related to the dimensions of dissolved methylcellulose chains, it provides information on the morphology and handling characteristics of the material prior to dissolution. The measurement settings are summarized in Table S1.

Table S1. Input measurement parameters used for particle size and shape analysis.

Parameter	Value
Pressure	600 mbar
Measurement time	30 s
Feeding mode	Manual
Analysis mode	General
Feeding rate	60%
Magnifications	0.3x and 4x
Acquisition mode	Automatic
Number of measurements	3

Figure S1a shows the volume-based equivalent diameter (xA) distribution of the methylcellulose powder. The characteristic particle size percentiles were Q10 = 62.0 µm, Q50 = 128.4 µm, and Q90 = 214.1 µm, corresponding to a median equivalent diameter of 128.4 µm. Representative particle images, classified according to equivalent diameter (xA) and aspect ratio, are presented in Figure S1b. The powder exhibited a broad particle size distribution and predominantly irregular particle morphology, which is typical of commercially milled methylcellulose powders.

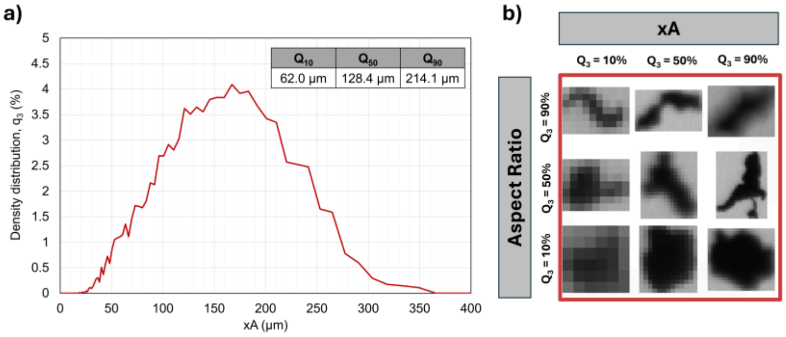

Figure S1. Particle size and shape characterization of the methylcellulose powder by dynamic image analysis (Litesizer DIA 500, Anton Paar) using a Dry Jet dispersion unit. (a) Volume-based particle size distribution expressed as the equivalent diameter (xA). (b) Representative particle images classified according to equivalent diameter (xA) and aspect ratio, illustrating the irregular morphology of the powder.
